# Progressive fibrosing interstitial lung disease in rheumatoid arthritis: A retrospective study

**DOI:** 10.3389/fmed.2022.1024298

**Published:** 2022-11-30

**Authors:** Anna Denis, Monique Henket, Marie Ernst, Nathalie Maes, Marie Thys, Céline Regnier, Olivier Malaise, Anne-Noëlle Frix, Fanny Gester, Colin Desir, Paul Meunier, Renaud Louis, Michel Malaise, Julien Guiot

**Affiliations:** ^1^Department of Pneumology, CHU of Liège, Liège, Belgium; ^2^Department of Biostatistics and Medico-Economic, CHU of Liège, Liège, Belgium; ^3^Department of Rheumatology, CHU of Liège, Liège, Belgium; ^4^Department of Radiology, CHU of Liège, Liège, Belgium

**Keywords:** interstitial lung disease, lung fibrosis, rheumatoid arthritis, epidemiological characteristics, disease progression, survival

## Abstract

**Background and objective:**

Rheumatoid arthritis associated-interstitial lung disease (RA-ILD) is the most common pulmonary manifestation of rheumatoid arthritis (RA) and an important cause of mortality. In patients suffering from interstitial lung diseases (ILD) from different etiologies (including RA-ILD), a significant proportion is exhibiting a fibrotic progression despite immunosuppressive therapies, defined as progressive fibrosing interstitial lung disease (PF-ILD). Here, we report the frequency of RA-ILD and PF-ILD in all RA patients’ cohort at University Hospital of Liège and compare their characteristics and outcomes.

**Methods:**

Patients were retrospectively recruited from 2010 to 2020. PF-ILD was defined based on functional, clinical and/or iconographic progression criteria within 24 months despite specific anti-RA treatment.

**Results:**

Out of 1,500 RA patients, about one third had high-resolution computed tomography (HRCT) performed, 89 showed RA-ILD and 48 PF-ILD. RA-ILD patients were significantly older than other RA patients (71 old of median age vs. 65, *p* < 0.0001), with a greater proportion of men (46.1 vs. 27.7%, *p* < 0.0001) and of smoking history. Non-specific interstitial pneumonia pattern was more frequent than usual interstitial pneumonia among RA-ILD (60.7 vs. 27.0%) and PF-ILD groups (60.4 vs. 31.2%). The risk of death was 2 times higher in RA-ILD patients [hazard ratio 2.03 (95% confidence interval 1.15–3.57), *p* < 0.01] compared to RA.

**Conclusion:**

We identified a prevalence of PF-ILD of 3% in a general RA population. The PF-ILD cohort did not seem to be different in terms of demographic characteristics and mortality compared to RA-ILD patients who did not exhibit the progressive phenotype yet.

## Introduction

Rheumatoid arthritis (RA) is a systemic inflammatory disease affecting 0.5–1% of the general population (1, 2). Through the development of biologic or Janus kinase inhibitor therapies, the joint prognosis of RA patients has improved significantly (3). Nevertheless, some extra-articular manifestations, including pulmonary involvement, are a major contributor to morbidity and mortality (4–6). Interstitial lung disease (ILD), referred to as rheumatoid arthritis-associated interstitial lung disease (RA-ILD), is the most common pulmonary manifestation (6–8). Understanding its pathogenic and clinical characteristics is crucial because no specific strategic therapy has not been established yet and patients remain difficult to treat (3). A low disease activity in the joints could prevent the emergence, progression and exacerbation of RA-ILD (3). It typically develops in the fifth or sixth decade and can be diagnosed up to 10 years after RA but sometimes occurs before joint symptoms (4, 9–12). According to the PERSEIDS study, it’s prevalence in Europe ranges from 1 to 18.1 per 10^5^ persons and among all subtypes of ILDs that are non-idiopathic pulmonary fibrosis (IPF), RA-ILD had the highest incidence in Belgium in 2018 (13).

More generally, in patients suffering from ILDs (including RA-ILD), a significant proportion is exhibiting a fibrotic progression despite appropriate treatment and regardless of the underlying ILD, defined as progressive fibrosing interstitial lung disease (PF-ILD) (14–16). Clinical, radiological and prognostic similarities are described with IPF which is the archetype of PF-ILD: accelerated respiratory failure, frequent exacerbations and early mortality (14, 16).

In 2019, the INBUILD trial used arbitrary criteria for progression of ILD within 24 months before screening, despite standard treatment with an agent other than nintedanib or pirfenidone (17):

– a relative decline in the forced vital capacity (FVC) of at least 10% of the predicted value;
– or a relative decline in the FVC of 5% to less than 10% of the predicted value and worsening of respiratory symptoms or an increased extent of fibrosis on high-resolution computed tomography (HRCT) of the chest;
– or worsening of respiratory symptoms and an increased extent of fibrosis on HRCT.

Among a global RA patients’ cohort, this retrospective study investigates the frequency and compares the characteristics and mortality rates of RA-ILD (vs. other RA) patients and PF-ILD (vs. other RA-ILD) patients.

## Materials and methods

### Population study

Patients were retrospectively recruited from our ambulatory care policlinic at University Hospital of Liège in Belgium from 01-01-2010 to 01-01-2020 based on a systematic evaluation of electronic hospital record using specific key word (RA). We selected patients suffering from RA according to ACR/EULAR 2010 Classification Criteria for Rheumatoid Arthritis (18). RA-ILD was defined as patients experiencing lung fibrosis of at least 10% of the lung parenchyma based on a systematic review by pneumologists on available HRCT of the chest.

Among RA-ILD patients, PF-ILD was defined according to INBUILD criteria [see “Introduction” section (17)]. Disease progression was considered at every hospital visit, using overlapping windows of 24 months prior to each hospital visit, until the first event meeting the definition criteria for progression was confirmed (after exclusion of other possible causes of progression: acute decompensated heart failure, bacterial or viral infection and/or ILD acute exacerbation). The end of follow-up was defined as the date of last follow-up visit, death, or lung transplant.

Age was defined at time *T* (i.e., the latest date on which patient underwent, in order of preference: A HRCT of the chest, a pulmonary function test (PFT) or a pneumology or rheumatology consultation). Biological values were the values occurring closest to time *T*. PFT values were the latest available. Treatments were defined at time *T*.

### Statistics

Qualitative variables were described using frequency tables, while continuous quantitative variables were described using statistical summaries (median and interquartile range).

Simple logistic regression models were performed. For each model, *p*-values were reported. If the odds ratios of the simple logistic regressions could not be calculated directly, a Haldane correction was performed and the *p*-value of the Fisher exact test was provided.

In order to allow for certain analyses, a logarithmic normalization of the PFT and biological data was performed. More precisely, a classical log transformation was performed for leukocytes (10^3^/mm^3^), platelets (10^3^/mm^3^) and fibrinogen (g/L) and a translational log(– + 0.1) was applied to measurement of CRP (mg/L).

Overall, survival was represented since the first available PFT by a Kaplan-Meier curve, compared between considered populations by Log-Rank test and supported by the Hazard Ratio (Manted-Haenszel).

Results were considered significant at the 5% uncertainty level (*p* < 0.05). The calculations were performed using SAS version 9.4 and the figures using Matlab 2019b.

No external funding was obtained and the study was conducted by clinicians on their own time.

The protocol was approved by the ethics committee of University Hospital of Liège (Belgian Number: B707201422832; ref: 2022/52).

## Results

**FIGURE 1:**
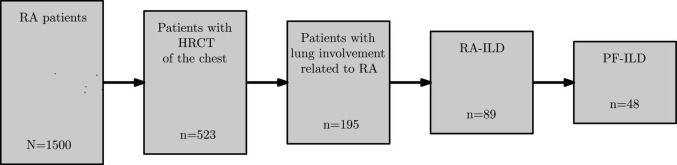
Flowchart of RA cohort. RA, rheumatoid arthritis; HRCT, high-resolution computed tomography; RA-ILD, rheumatoid arthritis associated-interstitial lung disease; PF-ILD, progressive fibrosing interstitial lung disease.

According to Figure 1, we identified out of a global cohort of 1,500 RA patients, 523 (34.9%) patients with at least one available HRCT of the chest, 195 (13.0% of 1,500) patients exhibiting a significant lung involvement associated with RA and RA-ILD in 89 cases (5.9%). More than half of the latter (48 patients, 53.9%) fulfilled the definition of PF-ILD.

Subjects’ demographic, pulmonary functional, biological and treatment characteristics are listed in Tables 1, 2.

**TABLE 1:** Comparison of patients’ characteristics between RA-ILD (*n* = 89) and other RA patients (*n* = 1,411).

	RA-ILD *n* = 89	Other RA patients *n* = 1,411	*P*-values
Age, years	71 (66–76)	65 (55–74)	**<0.0001**
Gender (male/female), n	41/48	374/1,037	**<0.0001**
Smoking history, %	70.9	63.1	**0.0014**
TLC ^a^, %pred	82.0 (64.0–94.0)	97.0 (82.0–106.5)	**<0.0001**
FVC ^a^, %pred	83.0 (66.0–98.0)	91.0 (78.0–104.0)	**0.0077**
FEV1 ^a^, %pred	80.0 (61.5–98.0)	86.0 (68.0–98.0)	0.31
DLCO ^a^, %pred	52.5 (39.5–62.5)	66.0 (50.0–79.0)	**<0.0001**
DLCO/VA ^a^, %pred	76.5 (62.0–87.5)	83.0 (66.0–94.5)	**0.013**
Hemoglobin, g/dl	12.70 (11.8–14.1)	13.20 (11.8–14.1)	0.47
Platelet count, 0.103/mm^3^	274.0 (212.0–334.0)	265.0 (217.0–330.5)	0.61
Leukocyte count, 0.103/mm^3^	8.87 (6.8–11.5)	7.47 (5.9–9.9)	**0.0008**
Lymphocytes, %	18.4 (11.0–29.1)	25.6 (16.4–32.8)	**0.031**
Monocytes, %	7.3 (5.7–9.3)	7.70 (5.80–10.0)	0.16
Neutrophiles, %	69.3 (55.5–79.6)	62.0 (53.6–72.8)	**0.017**
CRP, mg/l	8.9 (1.6–33.7)	4.1 (1.3–18.6)	**0.023**
Fibrinogen, g/l	3.9 (3.1–5.6)	3.9 (3.1–5.1)	0.93
ACPA positivity ^b^, n	50	665	0.72
Rheumatoid Factor positivity ^c^, n	50	602	**0.011**
Erosive RA ^d^, n	27	479	0.16
Treated with oral corticosteroids^e^, n	26	353	0.28
Treated with methotrexate^f^, n	25	578	/
Treated with leflunomide^g^, n	1	75	/
Treated with tumor necrosis factor-alpha inhibitors^g^, n	15	260	/
Treated with rituximab^g^, n	3	33	/
Treated with tocilizumab^g^, n	3	24	/
Treated with abatacept^g^, n	2	37	/

Continuous variables are expressed as median (interquartile ranges).

^*a*^At least one PFT was available for 79 RA-ILD patients and 375 other RA patients.

^*b*^Information about ACPA was available for 67 RA-ILD patients and 1,072 other RA patients.

^*c*^Information about rheumatoid factor was available for 72 RA-ILD patients and 1,117 other RA patients.

^*d*^Information about erosive RA was available for 33 RA-ILD and 657 other RA patients.

^*e*^Information about oral corticosteroids was available for 69 RA-ILD and 1,122 other RA patients.

^*f*^Information about methotrexate was available for 40 RA-ILD and 816 other RA patients.

^*g*^Information about leflunomide, tumor necrosis factor-alpha inhibitors, rituximab, tocilizumab and abatacept was available for 37 RA-ILD and 752 other RA patients. RA-ILD, rheumatoid arthritis-associated interstitial lung disease; RA, rheumatoid arthritis; TLC, total lung capacity; %pred,% of predicted value; FVC, forced vital capacity; FEV1, forced expired volume in 1 s; DLCO, diffusion lung capacity for carbon monoxyde; DLCO/VA, DLCO/alveolar ventilation; g/dl, grams per deciliter; mm^3^, cubic millimeter; mg/l, milligram per liter; g/l, gram per liter; ACPA, anti-citrullinated peptide antibodies; U/ml, units per milliliter; U/l, units per liter. Bold values mean *p*-value < 0.05.

**TABLE 2:** Comparison of patients’ characteristics between PF-ILD (*n* = 48) and non-PF-ILD patients (*n* = 41) among RA-ILD (*n* = 89).

	PF-ILD *n* = 48	Non-PF-ILD *n* = 41	*P*-value
Age, years	72 (66–77)	70 (65–75)	0.43
Gender (male/female), n	23/25	18/23	0.71
Smoking history, %	79.5	60.0	0.26
TLC^a^, %pred	83.0 (65.0–96.0)	78.0 (63.0–88.0)	0.76
FVC^a^, %pred	81.0 (66.0–97.0)	84.0 (70.0–99.0)	0.75
FEV1^a^, %pred	80.0 (65.0–92.0)	80.0 (61.0–98.0)	0.58
DLCO^a^, %pred	53.0 (37.0–62.0)	52.0 (41.0–63.0)	0.74
DLCO/VA^a^, %pred	78.0 (62.0–87.0)	70.0 (58.0–96.0)	0.98
Hemoglobin, g/dl	13.00 (11.80–14.40)	12.5 (11.2–13.0)	0.08
Platelet count, 0.103/mm^3^	253.0 (195.0–301.0)	310.5 (228.0–418.0)	**0.03**
Leukocyte count,0.103/mm^3^	8.15 (6.6–12.3)	9.14 (7.43–11.45)	0.44
Lymphocytes, %	22.7 (13.2–30.6)	16.9 (10.3–26.0)	0.59
Monocytes, %	7.30 (5.8–10.2)	7.35 (4.7–8.8)	0.46
Neutrophiles, %	67.1 (53.9–79.3)	72.1 (58.4–79.7)	0.60
CRP, mg/l	8.40 (1.5–28.8)	11.6 (2.4–63.9)	0.33
Fibrinogen, g/l	3.71 (3.1–5.4)	4.1 (3.0–5.9)	0.25
ACPA positivity^b^, n	29	21	0.87
Rheumatoid Factor positivity^c^, n	29	21	0.95
Erosive RA^d^	17	10	0.88
Treated with oral corticosteroids^e^, n	16	10	0.64
Treated with methotrexate^f^, n	12	13	/
Treated with leflunomide^g^, n	0	1	/
Treated with tumor necrosis factor-alpha inhibitors^g^, n	11	5	/
Treated with rituximab^g^, n	3	0	/
Treated with tocilizumab^g^, n	3	0	/
Treated with abatacept^g^, n	1	1	/

Continuous variables are expressed as median (interquartile ranges).

^*a*^At least one PFT was available for all 48 PF-ILD patients and for 31 non-PF-ILD patients. 10 patients were suffering from stable RA-ILD without symptomatic evolution or CT scan evolution and were included based on the clinical assessment. No PFT was available for this sub-cohort.

^*b*^Information about ACPA was available for 39 PF-ILD patients and 28 non-PF-ILD patients.

^*c*^Information about rheumatoid factor was available for 41 PF-ILD patients and 31 non-PF-ILD patients.

^*d*^Information about erosive RA was available for 21 PF-ILD patients and 12 non-PF-ILD patients.

^*e*^Information about oral corticosteroids was available for 40 PF-ILD and 29 non-PF-ILD patients.

^*f*^Information about methotrexate was available for 23 PF-ILD and 17 non-PF-ILD patients.

^*g*^Information about leflunomide, tumor necrosis factor-alpha inhibitors, rituximab, tocilizumab and abatacept was available for 22 PF-ILD and 15 non-PF-ILD patients. PF-ILD, progressive fibrosing interstitial lung disease; TLC, total lung capacity; %pred, % of predicted value; FVC, forced vital capacity; FEV1, forced expired volume in 1 s; DLCO, diffusion lung capacity for carbon monoxyde; DLCO/VA, DLCO/alveolar ventilation; g/dl, grams per deciliter; mm^3^, cubic millimeter; mg/l, milligram per liter; g/l, gram per liter; ACPA, anti-citrullinated peptide antibodies; U/ml, units per milliliter; U/l, units per liter; RA, rheumatoid arthritis. Bold values mean *p*-value < 0.05.

### Subjects’ demographic characteristics

Compared to other RA patients, RA-ILD patients were significantly older (65 years old of median age vs. 71, *p* < 0.0001), with a greater proportion of men (27.7 vs. 46.1%, *p* < 0.0001) and a higher percentage of smoking history. There were no statistically significant demographic differences regarding age, gender and smoking status between RA-ILD and PF-ILD cohorts.

### Pulmonary function tests

Spirometric values were lower than predicted values for all cohorts.

Other RA patients’ cohort had significantly higher total lung capacity (TLC) (97.0 vs. 82.0% expressed as predicted values, *p* < 0.0001), diffusing lung capacity for carbon monoxide (DLCO) (66.0 vs. 52.5% expressed as predicted values, *p* < 0.0001), DLCO/alveolar ventilation (76.5 vs. 83% expressed as predicted values, *p* = 0.013) and FVC compared to RA-ILD patients (91.0 vs. 83.0% expressed as predicted values, *p* = 0.0077).

Of interest, there was no significant difference with regard to forced expiratory volume in the first second (FEV1) between those two cohorts.

There were no statistically significant functional differences regarding PFT values mentioned above between RA-ILD and PF-ILD cohorts.

Criteria for disease progression and PFT relative changes within 24 months in PF-ILD patients are listed in Supplementary Table 1.

### Biological values

Compared to other RA patients, RA-ILD patients were exhibiting an increased leukocyte count (8 870/mm^3^ vs. 7 470/mm^3^, *p* = 0.0008), with a higher proportion of neutrophils (69.3 vs. 62%, *p* = 0.017), a lower percentage of lymphocytes (18.4 vs. 25.6%, *p* = 0.031) and a higher CRP value (8.9 mg/l vs. 4.1 mg/l, *p* = 0.023). Rheumatoid factor (RF) was more frequently positive in RA-ILD patients (602/1,117 vs. 50/72 patients, *p* = 0.011) while there was no difference concerning anti-citrullinated protein antibodies (ACPA).

Concerning PF-ILD and other RA-ILD patients’ comparison, the only biological significant difference was a lower platelet count in PF-ILD patients (253.10^3^/mm^3^ vs. 310.5.10^3^/mm^3^).

### Treatment characteristics

Although treatments were not recorded for all patients, use of corticosteroids, methotrexate and biological therapies (tumor necrosis factor alpha inhibitors, rituximab, tocilizumab and/or abatacept) reported equally among RA-ILD patients (26, 25, and 23 patients, respectively). In PF-ILD patients, biological therapies were reported more often than corticosteroids and methotrexate (18 vs. 16 and 12 patients, respectively), whereas in other RA patients, methotrexate was the most frequent treatment (578 patients vs. 353 on corticosteroids and 429 on biological therapies).

### High-resolution computed tomography analysis

**FIGURE 2:**
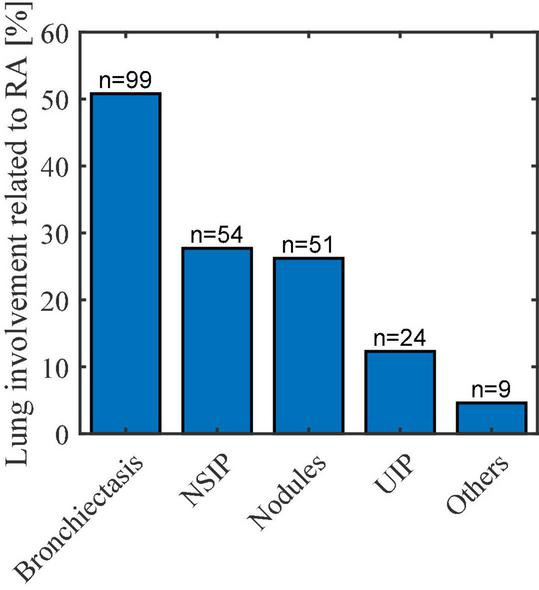
Lung involvement related to RA. NSIP, non-specific interstitial pneumonia; UIP, usual interstitial pneumonia.

According to Figure 2, the most frequent lung involvement thought to be associated with RA was bronchiectasis (up to 50% of all patients with lung involvement), followed by non-specific interstitial pneumonia (NSIP) pattern (27.7%), lung nodules (26.2%), usual interstitial pneumonia (UIP) pattern (12.3%), and others (4.6%).

ILD patterns in RA-ILD and PF-ILD groups are listed in Table 3. Among RA-ILD patients, NSIP pattern was the most frequent (60.7%) followed by UIP (27.0%) and other or mixed patterns (12.6%). In PF-ILD patients, NSIP was also the most frequent pattern (60.4 vs. 31.2% for UIP and 8.3% for mixed or other patterns).

**TABLE 3:** Interstitial lung disease patterns in RA-ILD (*n* = 89) and PF-ILD patients (*n* = 48).

	RA-ILD *n* = 89	PF-ILD *n* = 48
NSIP, n (%)	54 (60.7%)	29 (60.4%)
UIP, n (%)	24 (27.0%)	15 (31.2%)
Mixed pattern or other pattern, n (%)	11 (12.6%)	4 (8.3%)

RA-ILD, rheumatoid arthritis-associated interstitial lung disease; PF-ILD, progressive fibrosing interstitial lung disease; NSIP, non-specific interstitial pneumonia; UIP, usual interstitial pneumonia.

### Survival analysis

**FIGURE 3:**
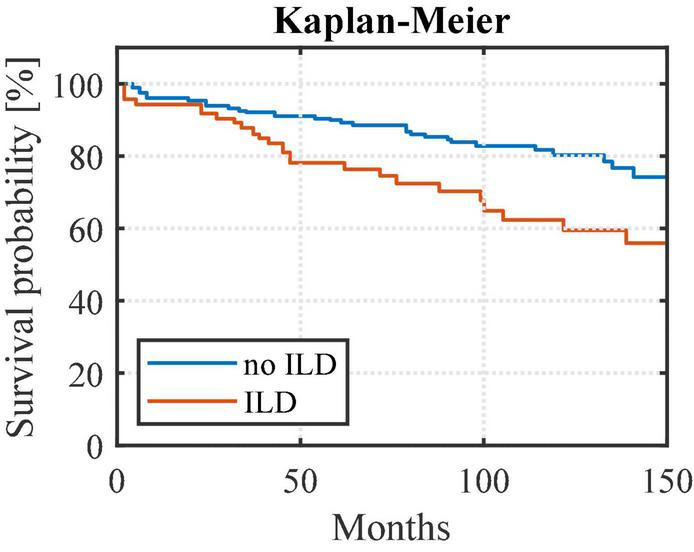
Survival curves from first available pulmonary function test comparing RA-ILD to other RA patients. Rheumatoid arthritis-associated interstitial lung disease (ILD) patients exhibited higher mortality rates than rheumatoid arthritis patients without ILD (noILD) (*p* < 0.01). noILD, no interstitial lung disease; ILD, interstitial lung disease.

RA-ILD patients exhibited higher mortality rates than RA patients without ILD (*p* < 0.01) (Figure 3). The risk of death was 2 times higher among the RA-ILD group [hazard ratio 2.03 (95% confidence interval (CI) 1.15–3.57)] compared to RA patients. The relevance of the statistical evaluation comparing mortality between RA-ILD and PF-ILD groups was considered to be non-significant as only 9 patients died in the PF-ILD group.

## Discussion

A progressive phenotype was observed in approximately 3% of a global RA cohort and in up to 50% of an RA-ILD cohort in this retrospective study.

RA-ILD patients were older than the general RA population with a male predominance and an increase in tobacco exposure. Confirmatory, previous studies already identified that RA-ILD typically develops in the fifth or sixth decade and the male-to-female ratio may be as high as 2:1 in some studies (whereas RA-non-ILD more often occurs in women) (4, 9, 10, 19).

Cigarette smoking has been shown to increase expression of the enzyme responsible for citrullination in the lungs, transforming arginine into citrulline and creating new epitopes against which autoantibodies, called anti-citrullinated protein antibodies (ACPA) can react (20, 21). In a large Swedish cohort study, smoking and having a double copy of the shared HLA-DR epitope increased the risk of RA by 21-fold compared to patients without this combination (22). This suggests that, in case of a genetic predisposition, smoking may promote anti-citrulline autoimmunity, a possible pathophysiological mechanism for the development of RA and RA-ILD (5).

Six percent of RA patients involved in this study were suffering from RA-ILD. This prevalence is most likely underestimated because of the retrospective design of the study and the restricted number of patients with available chest HRCT. Although HRCT currently replaces lung biopsy in standard practice for the diagnosis of RA-ILD (23, 24), underdiagnosis probably persists because of the poor symptomatology and the lack of awareness of clinicians regarding RA-ILD (23). Symptoms may also be masked by the lack of exercise performed by RA patients due to their joint symptoms or due to steroids induced myopathy (23). Studies estimate that 45–68% of patients show involvement on HRCT or PFTs while only 10% have clinically active RA-ILD (25, 26). In our study, RA-ILD represented 17% of all RA patients with an available HRCT. A further limitation was that RA-ILD group was compared to the general RA population rather than only patients with an available HRCT because it was assumed that patients without HRCT were asymptomatic and therefore with a low probability of suffering from any lung disease. Besides, the systematic review of the HRCTs was performed by pneumologists (and not expert radiologists).

Among described HRCT patterns, UIP is usually the most described, reaching 40–65% of RA-ILD cases depending on the studies followed by NSIP, affecting approximately 10–40% of patients (10, 27). In disagreement with these findings, NSIP was more frequent in the present study among RA-ILD and PF-ILD patients (including 60.7 and 60.4% of cases, respectively), while UIP was found in 27 and 31.2% of patients. This observation is thought to be due to the restricted number of RA-ILD patients in our study.

Confirmatory to previous studies, RA-ILD patients showed significantly higher mortality rates than RA patients without ILD. According to Hyldgaard et al., RA-ILD patients die 2–10 times more than those without ILD (28). Other studies show that fibrotic lung involvement is responsible for 10–13% of RA-associated mortality, making it the second leading cause of death after cardiac involvement and that mean survival times vary from 2.6 to 10 years (4, 29–31). This high mortality rate, together with the fact that most patients present subclinical findings, raises the question of early screening for ILD signs among RA patients.

Moreover, UIP pattern is generally associated with a more severe prognosis compared to NSIP (32, 33). However, according to Solomon et al., regardless of the UIP or NSIP pattern, patients with a 10% decline in FVC (% predicted value) had an increased risk of mortality, implying an increased risk of death in case of functional decline defined as a 10% decline in FVC (30).

Nearly 50% of RA-ILD patients showed a PF-ILD phenotype in the present study. This percentage is higher than in the PERSEID study, a large European retrospective study, demonstrating a progressive pattern in 38% of RA-ILD patients (13). We believe that screening PF-ILD patients is of potential interest for the therapeutic management of RA-ILD patients. Treatment of RA-ILD is currently mainly based on retrospective studies due to the absence of randomized controlled trials studying the impact of RA specific therapies on the evolution of ILD in comparison to the standard of care (4, 27, 34). Historically, it was recognized that drug-related pulmonary toxicity could be a confounding factor in RA whereas several studies suggested that this was previously over-estimated (27, 35).

In the particular context of PF-ILD, regardless of the underlying etiology, parenchymal fibrotic changes seen in patients suffering from progressive ILD are thought to have common mechanisms of self-reinforcing progressive fibrosis (including impaired cellular repair, fibroblast proliferation, and alveolar dysfunction) (14, 15, 18). This calls for a homogeneous therapeutic strategy using, in particular, antifibrotics drugs which demonstrated their efficacy in IPF, the archetype of PF-ILD (14–16, 31). Confirmatory, the INBUILD trial, a double-blind, placebo-controlled, phase 3 trial conducted in 15 countries, showed that patients suffering from PF-ILD were presenting a significant reduction of lung decline over time (17). RA-ILD patients, especially with the UIP pattern, share common epidemiologic, clinical, genetic and radiologic features with IPF patients (4, 27, 34, 36, 37) increasing the rationale of using anti-fibrotic therapies in this particular subgroup. Genetic similarities regarding variant of the MUC5B promoter known to be a major risk factor for IPF have been found in RA-ILD: Juge et al. observed that this variant was more frequent in RA-ILD than in unaffected controls (adjusted odds ratio, 3.8; 95% CI, 2.8–5.2; *p* = 9.7 × 10^–17^) (38, 39).

The use of antifibrotic therapies in RA-ILD (and especially PF-ILD) is further supported by a mouse model in 2018 showing a reduction of both fibrosis and joint disease after nintedanib use in RA-ILD mice (40). In addition, pirfenidone reduces levels of interleukine-6 and tumor necrosis factor alpha, two cytokines involved in the pathogenesis of RA (41). Wu et al. showed that pirfenidone inhibited fibroblast to myofibroblast transition in lung fibroblasts from RA-ILD patients (42).

The INBUILD study showed that the FVC decline over 52 weeks was significantly lower in patients with PF-ILD of various origins (except IPF) treated with nintedanib compared with placebo. In the overall population, the adjusted rate of decline in the FVC was –80.8 ml per year with nintedanib and –187.8 ml per year with placebo, for a between-group difference of 107.0 ml per year (95% CI, 65.4–148.5; *p* < 0.001) (17). A *post hoc* subgroup analysis suggested a benefit of treatment in terms of slowing the decline of FVC in all PF-ILD subgroups, including connective tissue disease-related interstitial lung disease (CT-ILD) (and involving 89 patients with RA-ILD) (43).

In the future, randomized controlled trials are necessary in order to study the efficacy of these antifibrotic therapies in RA-ILD and to determine valid screening tools. As for screening some complications in other autoimmune diseases (e.g., bone remodeling markers in systemic scleroderma), various biomarkers could be of interest in order to identify RA-ILD patients at risk of progression but still need to be thoroughly validated before being implemented in clinical use (44, 45).

For the first time, American Thoracic Society (ATS) published guidelines for “progressive pulmonary fibrosis” (PPF) definition in 2022 (46). They define PPF in patients meeting at least two of the three following criteria occurring within the last year in the absence of alternative explanation: worsening of respiratory symptoms, physiological evidence of disease progression (absolute decline in FVC of > 5% or absolute decline in DLCO of > 10%) and radiological evidence of disease progression. The decision was made to maintain the use of INBUILD criteria for “PF-ILD group” in the present study as it was conducted prior to the ATS publication (17). These ATS guidelines suggest the use of nintedanib in patients who have failed standard management for fibrotic ILD, other than IPF, by referring mainly to INBUILD study and its *post hoc* analysis as evidence, implying that the use of its inclusion criteria are in line with the therapeutic possibilities in these patients (17, 46). Meanwhile, the 2022 ATS definition should be considered in future prospective studies.

## Conclusion

In conclusion, the current study provides valuable information about RA-ILD and PF-ILD patients in a single-center academic cohort of patients suffering from RA. These results show a 6% prevalence of RA-ILD with half of them presenting a progressive phenotype. While RA-ILD have a higher mortality rate and are mainly older men with lower PFT values and higher smoking status and CRP values compared to other RA patients, there were no differences concerning survival and other characteristics between RA-ILD and PF-ILD patients. There remains an unmet need to identify at the earliest stage patients suffering from the progressive phenotype with a specific screening and therapeutic strategy based on further dedicated large clinical trials.

## Data availability statement

The raw data supporting the conclusions of this article will be made available by the authors, without undue reservation.

## Ethics statement

The studies involving human participants were reviewed and approved by the Ethics Committee of University Hospital of Liège (Belgian Number: B707201422832; ref: 2022/52). Written informed consent for participation was not required for this study in accordance with the national legislation and the institutional requirements.

## Author contributions

AD was the lead writer of the original draft and contributed equally to others to conceptualization (ideas; formulation or evolution of overarching research goals and aims), data curation, investigation, methodology and visualization (preparation, creation, and presentation of the work). MH contributed equally to others to conceptualization, data curation, formal analysis, investigation, methodology, validation (verification of the overall replication and reproducibility of the results and the other research outputs), and review of writing. ME, NM, and MT contributed equally to data curation, formal analysis, investigation, and methodology. ME contributed to review of writing. CR, OM, A-NF, FG, CD, and PM contributed equally to review of writing. FG contributed equally to others to data curation and investigation. RL and MM contributed equally to supervision and review of writing. JG was leader in conceptualization, methodology, resources and review of writing and contributed equally to investigation, supervision, and validation. All authors contributed to the article and approved the submitted version.

## Abbreviations

ACPA: anti-citrullinated peptide antibodies
ATS: American Thoracic Society
CI: confidence interval
CRP: C-reactive protein
CTD-ILD: connective tissue disease-related interstitial lung disease
DLCO: diffusing lung capacity for carbon monoxide
FEV1: forced expiratory volume in the first second
FVC: forced vital capacity
HRCT: high-resolution computed tomography
ILD: interstitial lung disease
IPF: idiopathic pulmonary fibrosis
NSIP: non-specific interstitial pneumonia
RA: rheumatoid arthritis
RA-ILD: rheumatoid arthritis-associated interstitial lung disease
PF-ILD: progressive fibrosing interstitial lung disease
PFT: pulmonary function test
RF: rheumatoid factor
TLC: total lung capacity
UIP: usual interstitial pneumonia.
