# Supplementary material for: Progressive fibrosing interstitial lung disease in rheumatoid arthritis: A retrospective study
Source: Front Med (Lausanne). 2022 Nov 30;9:1024298. doi: 10.3389/fmed.2022.1024298 (PMC9748274; doi:10.3389/fmed.2022.1024298)
Supplement: Supplementary file 1 [file Table_1.DOCX]

**Supplementary material**

**sTable 1. Criteria for disease progression and PFT relative changes within 24 months in PF-ILD patients (n=48)**

|  | **PF-ILD**  n = 48 |
| --- | --- |
| **Criteria for disease progression in previous 24 months**   - Relative FVC decline of ≥ 10% pred, n (%) - Relative FVC decline of 5-10% pred plus worsening of respiratory symptoms or increased extent of fibrosis on HRCT, n (%) - Worsening of respiratory symptoms and an increased extent of fibrosis on HRCT, n (%) | 31 (64.6%)  1 (2.1%)  16 (33.3%) |
| **PFT relative changes within 24 months**   - FVC relative changes, % - DLCO relative changes, % | -13.9 %  -16 % |

Abbreviations: PFT, pulmonary function test; PF-ILD, progressive fibrosing interstitial lung disease; FVC, forced vital capacity; HRCT, high resolution computed tomography; DLCO, diffusing lung capacity for carbon monoxide.
